# UNASUR Health: A quiet revolution in health diplomacy in South America

**DOI:** 10.1177/1468018115599818

**Published:** 2015-12

**Authors:** María Belén Herrero, Diana Tussie

**Affiliations:** Facultad Latinoamericana de Ciencias Sociales (FLACSO), Argentina

**Keywords:** Global, regional health diplomacy, regional UNASUR, South America, social policy

## Abstract

Since the creation of Union of South American Nations (UNASUR), health policies became a strategic factor in South America to collectively balance the legacy of neoliberal policies in the region. The aim of this article is first to describe the social, political, and economic processes that explain the emergence of UNASUR and its focus on social policy through healthcare. We then analyze how by virtue of *UNASUR’s Health Council*, healthcare became the spearhead of cooperation giving way to novel forms of diplomacy. In so doing, this article contributes to a broader understanding of the regional health diplomacy and the process of *unasurization* of health policies as the process of building a new health framework.

## Introduction: Region building in South America after neoliberalism

The Union of South American Nations (UNASUR) is part of an ambitious policy agenda of transformative regionalism (Riggirozzi, 2014b). Its emergence needs to be placed in the wider context of the evolution of regional governance. It emerges from a consistent effort in the region to broaden the scope of its institutions beyond trade. In an altered context, content also changes. Much of UNASUR’s initial momentum stemmed from the rejection of some leaders to earlier neoliberal attempts at market opening regional integration and the regional role of the United States. When studying the spread of neoliberalism, much has been said about the role of international economic institutions –the World Bank, the International Monetary Fund, and World Trade Organization – in inducing countries into acceptance of deregulation and rules for opening markets. Yet in Latin America, the project of the Free Trade Area of the Americas was a leading hand of the Washington Consensus and just as commanding as the global institutions. Trade agreements have long standing consequences insofar as they are treaties with quasi permanent commitments, in contrast to the conditionality lending of multilateral banks which is over once the loan is repaid.

As Mace and Bélanger (1999) have pointed out (p. 42), the only counterbalance to the US predominance in this particular continent is collective action. The spirit of UNASUR, in other words, diverged from the US-dominated hemispheric mold of cooperation that informed the Organization of American States (OAS) and the Inter-American Treaty of Reciprocal Assistance during the Cold War, as well as from post-Cold War initiatives inspired by neoliberal open-regionalism, such as the Southern Common Market (MERCOSUR) or the North American Free Trade Agreement (NAFTA).

UNASUR comes as part of the new cycle of politicization in regional politics (Dabene, 2012) or as Riggirozzi and Tussie (2012) posit, as part of a struggle for post-hegemonic regionalism. What makes UNASUR particularly interesting is its vision of regionalism that builds from, and capitalizes on, pre-existing trade-led agreements, specifically MERCOSUR and the Andean Community, but strengthens new areas of regional cooperation, way beyond the trade glue. The emergence of UNASUR is an interesting case to analyze changes in the form and content of regional governance. Very quickly it acquired a central role, though naturally not at all an uncontested one, in regional governance. The possibility of intensifying contacts among presidents was one of the central motivations behind the creation of UNASUR (Sánchez and Hoffmann, 2014). The presidents’ focus was to build a platform for flexible and pragmatic cooperation focusing on infrastructure, health, security, and natural resources. Presidential empowerment to such an extent then tried to legitimize itself by pluralization of the actors involved. Although the Council of Heads of States is preeminent in the building of the institution as such, it relies on a number of bodies to support decision making from meeting to meeting and provide institutional density to functional cooperation. Functional cooperation and policy coordination (as distinct from the political coordination at presidential level) has floundered in some areas such as finance (Trucco, 2012), whereas in health it has made remarkable inroads and provided stepping stones to institutional development and a novel diplomacy. The Constitutive Treaty of UNASUR establishes a broad acceptance of social policy as an important catalyst for new modes of cooperation and the need to institute a new regional body. What is more, of all the functional councils, the health council was the first to be created and remains the most active despite festering differences at other levels. As argued by Riggirozzi (2014b) the attention to health policies shows a ‘social turn’ in the life of regional organizations and their mission to cooperate in order to meet high profile social demands (p. 434). The ‘social turn’ reclaims the region for ‘positive regionalism’ that introduces rights and inclusion through regional policies (Scharpf, 1996). Research on regional public goods has overlooked health, while regional social policy analysis has not examined whether and how regional organizations’ policies are being implemented through social institutions (Deacon et al., 2009; Yeates and Deacon, 2006).

Health diplomacy and indeed South American regional social radicalism picked up on a series of global moves, the 1978 Alma Ata principle of ‘Health for all’, the Millennium Development Goals (MDGs) and the World Health Organization (WHO) Commission on Social Determinants of Health. The Commission, created in 2004, not only elevated efforts to improve health and revert the social causes of inequalities, but also reinforced the importance of international cooperation. This context enabled the multiplication of processes at all levels (multilateral, regional, and bilateral). That wave, together with the rise of left leaning administrations in a number of countries in South America allowed an opportunity structure for pioneering health diplomacy and social inclusion through health.

The article proceeds in three parts. We first describe the process of emergence of health diplomacy. We analyze the political and economic conditions that explain such emergence focusing on the legacy of neoliberal reforms. We consider this legacy to have laid out the conditions, and indeed the need, for a new form of health diplomacy in South America. We then turn our eye to UNASUR fleshing out the way commitments of social development were embraced and reflected in the health area. We argue that such developments were first inspired by the humanist perspective of presidents in power. As the process gained momentum, health remained largely, but not totally, above the political fray. It acquired a certain life of its own and strengthened the bonds between health and international diplomacy.

## The emergence of health diplomacy

Health is a prime example of an ongoing quiet revolution in the regional political economy of cooperation and diplomacy. Yet it is important to put present day regional health diplomacy in a wider historical context. The medical sciences as all sciences have a long tradition of being organized internationally and their global networks can be seen as instruments for sharing values and enhancing trust-building activities. But scientists are also very much organized at national levels and hence their activities can be seen by traditional diplomacy as tools for promoting national brands and pursuing economic goals (Solingen, 1994). In the emergence of health diplomacy, three pioneering Latin American conferences held in the second half of the 19th century stand out: the first two involved the Brazilian Empire and the Republics of Uruguay and Argentina, and were held in Montevideo in 1873, and in Rio de Janeiro in 1887; the third took place in Lima, Peru, in 1888, and included Bolivia, Chile, Ecuador, and the host country. While, in Europe, the international sanitary conferences served as the genesis of the WHO in 1948, in the Americas, the 1873 and 1887 conferences led to the establishment of the Pan American Health Organization (PAHO), the oldest international health agency in the world, born in 1902 as the International Sanitary Bureau. At the time the coastal cities of South America had become rather powerful magnets for massive migration flows, authorities became aware that they needed to rationalize the very diverse set of quarantine regulations they each held with a view to making them more effective and at the same avoid the disruption of their mutual trade, especially foodstuffs. Realizing that such challenges could only be faced jointly, an effort to coordinate health prevention gained ascendancy. The 1873 event held in Montevideo sought to standardize quarantine measures and related policies applied to vessels infected by cholera, yellow fever, and the plague. The first South American International Sanitary Conference sought the protection of a connected South America against what were seen as the ‘exotic epidemics’ which traveled with migrants. Participants in the conference were leading members of the generation of hygienists that began to hold public office, as part of a process that established and consolidated the medical profession in each country. The tie between physicians and the state became increasingly strong in the second half of the 19th century (Lima Chaves, 2013) and so were the links between international medicine and politics. The Pasteur mission to Brazil in 1901 played a critical part in developing, accelerating, and catalyzing pre-existing trends in that country, and in placing Brazil in the Latin American vanguard in research and control of various ‘tropical’ diseases.

Hence, in some respects, social policy through regionalism is hardly ‘new’ in South America. The difference between those early XIX century efforts and the XXI century ones is that the former grew out of apprehension and led to coordinated surveillance. They were preventive efforts *vis-a-vis* ‘exotic epidemics’ that arrived with the intense docking of vessels as a result of rapidly expanding international commercial ties. In contrast, the present efforts to regionalize social policy, as we shall see, reach out to offer a more positive agenda for wider social inclusion. But the long-term importance of those early efforts lies in the ties that brought together the medical profession with the political institutions within and beyond their countries to regulate trans-border health risks.

Global health governance solidified and diversified after World War II and new concepts and concerns appeared with the 1948 establishment of the WHO (Fidler, 2010). Some decades after, in Latin America in particular, the ‘movimiento sanitarista’ contributed to step up cooperation in the region (Gomez, 2012). Until the rise of military rule and the debt crises of the 1980s, South America, generally speaking, enjoyed relatively strong national welfare state formations in which social policies and health, in particular, were key axes. Health was conceived as a right and institutionalized (Tobar, 2001). In this sense, the right to health was linked to the concept of citizenship and the political construction of the nation states, while health, however erratically pursued, played a key role in the democratic ethos. In this trend, Latin America was a leading region in the promotion and practice of social medicine since 1960. It was swiftly reasserted when the process of re-democratization picked up (Mariani, 2007), but coetaneous to the green shoots of democratization, the international financial institution, and in particular the World Bank gained the upper hand in policy writ large. In the social sectors, the reform was meant to remove the idea of equity and universalism as an organizing principle for national social policy. Although these ideas were pushed aside when targeting gained the day, they remained the organizing principle of the movimiento sanitarista’s openly political strategy. Sanitarism and social medicine (especially in Brazil) continued to work to develop a linked approach to social epidemiology and to assert the ambitious goal of collective health and social determinants of health to address the causes of ill health: poverty and inequalities (Solar and Irwin, 2010; Whitehead et al., 2001). After the long neoliberal decade, their turn to reassert their influence came with the rise to power of a string of left leaning governments since the early XXI century. The opportunity structure was laid out to re-embed these rights in national policy and in a set of international institutions which might ensure that the global economy had a social or public purpose.

In this context, at the global level, for example, the MDGs were a welcome shot in the arm. Specifically, the eight MDG affirmed health as a focal point of global governance. Three of the MDGs target specific health objectives (HIV/AIDS, maternal health, and child health), and four others attempt to improve social determinants of health. The WHO Commission on Social Determinants of Health then injected further momentum. Fidler calls this moment the ‘revolution’ in global health to denote the increasing role of health in foreign policy (Fidler, 2010; Labonté and Gagnon, 2010). It has generated an unprecedented hike in funding and similarly growing influence of policymakers, activists, and philanthropists who claim health as a foreign policy issue of first-order importance. As a result, global health became an essential part in the equation of international relations (Fidler, 2001, 2010).

When trade-regional organizations were set up in the 1990s in South America, cross-border projects on regulations for health, education, and labor were embedded in both Andean Community and MERCOSUR. Art. 10 of the 1988 Additional Protocol to the American Convention on Human Rights in the Area of Economic, Social, and Cultural Rights had by then included access to health as a human right, governing the conduct of states, organizations, and individuals. The appeal to social economy and human development has lived in the collective imagination and even manifested in institutional forms. In the case of Andean Community, for instance, two managing bodies were created to manage common challenges in the areas of health and education. The Hipólito Unanúe agreement and the Andres Bello Convention established the foundations for the coordination of health and education policies respectively since the early 1970s. In the area of health, active policies in relation to the prevention and control of diseases affecting border areas were implemented, whereas in education, policies toward the harmonization of curricula, mobility of students and professionals, and quality assurance programs were set up for the Andean region (UNDP, 2011).

In MERCOSUR, a Meeting of Health Ministers was established in the mid-1990s to take up health issues emerging as a byproduct the trade agenda and mainly in relation to customs issues, or in other words, the health aspects of trade in goods. Similar advances were also seen in the area of education, which together with health pushed social policy into the agenda for activities and surveillance with some degree of impact in these fields (UNDP, 2011). Yet, further commitments and institutionalization of cooperation and implementation of social policies were haphazard and with mixed consequences for human development in both of these trade integration schemes during the 1990s. Altogether they were based on the belief that governments should only provide minimal or basic levels of social provision and social protection.

In practice, these embryonic regional social agendas were working on the edges of pro-market reforms and the financial duress. Actions plans remained somewhat lost in a haze of good intentions (Deacon et al., 2009). In this context, some commitments over a range of social policy areas were made but low levels of institutionalization for implementation, coordination, and compliance affected the depth and pace of regional social policies. Politically, rudimentary institutional structures for such issues in both Andean Community and MERCOSUR left social policy subject to the conditionality of international financial institutions and the unleashing of the business appetite of private providers.

In this sense, the open-regionalism of the neoliberal 1990s (the tag used to contrast it with the less market-oriented construction of past decades) understood Latin America as part of an Americanized system, looking to the north and postulated regionalism through financial and trade linkages (Grugel, 1996; Phillips, 2003; Tussie, 2009a). Global health policies were a central site for implementing neoliberal reforms, especially after the *World Development Report: Investing in Health* (World Bank, 1993) and hand in hand with the Free Trade Area of the Americas in the region. For one, the World Bank became increasingly interested in the reorganization of public sectors, including the health sector, and the Bank sharply increased its loans for health restructuring while private investment was welcomed. The World Bank also had traction over Inter-American Bank programs (Babb, 2009; Vivares, 2012) and together they called for cutting down public involvement in health services delivery and instructed many countries to reduce public expenditure on health. Second, they recommended the partial ‘cost-recovery’ of public health services by charging user fees. And third, they insisted on an increased reliance on the market to finance and deliver healthcare as well as calling for privatization of public healthcare services (Akin et al., 1987). Altogether, country programs shifted their priorities from programs designed to serve those most in need to programs that offered cost-effective interventions while throwing open the door to the business interests of international health insurance companies, such as AIG, AETNA, MetLife, Accord together with providers of equipment, medicines, and services. Market-making policies gained the day and healthcare became market driven as poverty rates and income inequality increased region wide (Riggirozzi, 2014b). Welfare provision was systematically squeezed and access reduced to those who could pay for healthcare services, medicines, and some good schooling. The shift toward selective primary healthcare and the primacy given to business interests generated rather acute controversy. The political complexities within the field of health are often defined by tensions between the interests of Big Pharma, private insurance providers, national health systems, and citizens’ access. These problems have certainly played a role in stimulating new actors to move into the health arena and to search for new institutional arrangements.

By the new millennium, Latin America became witness to a series of political transitions from the Right/Right of Center to Left/Left of Center. Governments committed to more democratic economic management, deeper and broader popular representation, redistribution, and better and more accessible public services took office in Venezuela in 1998, Brazil and Argentina in 2003, Uruguay in 2004, Bolivia in 2005, Ecuador in 2006, and Paraguay in 2008. The rise of what is known as the ‘New Left’ is an indication that the more cautious, consensual, and pro-elite democracies that characterized the early stages of democratization were coming to an end. Instead, claims abounded that Latin America’s political economy should be focused on the needs of ordinary and forgotten people of the region. This shift is often characterized as a move away from neoliberalism to post-neoliberalism (Grugel and Riggirozzi, 2009; Ruckert and MacDonald, 2009), reflecting more radical models of political inclusion and citizenship.

The New Left has been explained as a reaction against what came to be seen as excessive marketization and the elitist and technocratic democracies that accompanied market reforms at the end of the 20th century. By force, the profound changes in the political economic orientation in many countries in the region called for redefining the dynamics in region-building. The outside-in dynamic shifted to an inside-out one, whereby the domestic political economy was projected, and projected with a shift to trans-national solidarism. The move was not simply a domestic political swing to reach out to the excluded but also a window of opportunity for new leaders to synchronize governmental policies in the search for greater autonomy *vis-à-vis* external actors (Riggirozzi and Tussie, 2012).

These points are the key to understanding why an essentially political body as UNASUR takes on the politics of solidarity and health, in particular as a cementing issue for region building. The move was accompanied by an aversion to donor-driven agendas and the international NGO’s self-interest in winning donor contracts to substitute for government social services. The right to health, universal access, and social determinants became overtly political flags. In 2008, 12 heads of state and government signed the Treaty of the Union of South American Nations in order to

build a participatory and consensual manner, an integration and unity in the cultural, social, economic and political between their peoples, prioritizing political dialogue, social policies, education, energy, infrastructure, financing and the environment, among others, with a view to eliminating socioeconomic inequality, achieve social inclusion and civic participation strengthen democracy and reduce asymmetries within the framework of strengthening the sovereignty and independence of States. (UNASUR, 2009: 9)

As UNASUR came into being, leaders collectively challenged a view of development that reduced it to its trade and economic dimension. Social policy and health became one of the cornerstones of collective diplomacy. In particular, Brazil and Venezuela were foremost among those challenging the premises of the neoliberal health agenda. Together, the Brazilian and Venezuelan governments in response *to* or in alliance *with* civil society moved to create a consensus over selected topics and create a regional institution that could be operational and which could also lay the foundations for a body of thought. In consequence, regional diplomacy moved to de-neoliberalize and health diplomacy in particular became a strategic policy driver whereby joint efforts could flag a cooperative normative framework to counter the Washington Consensus.

In this context, health illustrates a strategic policy area where the social turn in regional cooperation searched for renewed collective goals, norms, and practices. The social turn in domestic policies was projected into regional cooperation that not only portrays a new mode of understanding what regional governance is about, but also the region’s interest in projecting social concerns in the international arena. South America became a space for contention and contestation, as well as an arena of consensus-building. In short, UNASUR Member States understood that the best way to enhance and coordinate efforts would be through the creation of intergovernmental sectoral councils composed of the respective ministers. The UNASUR Health Council became a site where new modes of practices redefined regional cooperation with a social purpose. In line with the argument laid out by Riggirozzi (2014a), we consider that this turned UNASUR into a central actor engaged in regional health diplomacy, understood as a double track process that covers regional and international cooperation.

Such unpacking of regions allows us to move away from one-dimensional views that posit regional cooperation as mainly led by the imperatives of the global economy (Tussie, 2009a). Thus, the creation of UNASUR is a space of socialization, for consensus building and the establishment of common interests. The trade dimension was scarcely mentioned in the treaty which makes clear the eminently political nature of the organization and the importance attached to cooperation.

## The building blocks in UNASUR

The way UNASUR embraced new commitments of social development was due in no small measure thanks to the pioneering efforts of Brazil and Venezuela. Both countries made huge leaps to strengthen the bonds between health and international diplomacy. On the part of Venezuela, the Miracle Missions follows in the steps of Cuban medical internationalism. The Missions consist of a concessional regional social program that grants free eye care. Founded in Cuba in 2004 with Venezuelan patients, the project was then relocated to Venezuela where it offers free care to needy patients from all over Latin America and the Caribbean. Almost 3 million surgeries have been performed tapping on Cuban medical expertise and putting Venezuela at the center of a rather spectacular people-to-people regional program. The case of Brazilian health diplomacy may be lower key but not less effective as an agenda setter. Brazil had a leading global role in the negotiations that led to the Framework Convention for Tobacco Control, the first treaty negotiated under the auspices of the WHO. Moreover, together with India and South Africa in the World Trade Organization, the threesome spearheaded the 2001 Doha Declaration on Intellectual Property and Public Health allowing the circumvention of patent rights for better access to essential medicines in the case of epidemics. The leading personality of Paolo Buss, as head of the Fiocruz Foundation^1^ from 2001 to 2008 and president of the World Federation of Public Health Associations, was a prime mover, as was former President Lula, a trade unionist and active participant in the World Social Forum together with the late President Chavez of Venezuela (Cepik and Sousa, 2011). Both Lula and Chavez not only had a heartfelt sense of social commitment to the dispossessed and vulnerable but also a flair for a deeply personalized diplomacy aiming to make contact with ordinary people and their needs. On the part of Venezuela, there was a commitment to medical international activism following closely on Cuba’s footsteps. Brazil could count on the political ascendancy of the *movimiento sanitarista* and the diplomatic knowhow of driving global processes (Tussie, 2009b).

In 2007, the foreign ministers of Brazil, France, Indonesia, Norway, Senegal, South Africa, and Thailand issued the Oslo Declaration identifying global health as ‘a pressing foreign policy issue of our time’ (Labonté and Gagnon, 2010: 1). The declaration hand in hand with the much publicized 2008 report of the Commission on the Social Determinants of Health reflects a 10-year trend in which health rose to prominence in global policy agendas. Such prominence sets the stage for the concept of global health diplomacy to describe ‘the processes by which government, multilateral and civil society actors attempt to position health in foreign policy negotiations and to create new forms of global health governance’ (Labonté and Gagnon, 2010: 1). As soon as UNASUR was launched a year later, its Health Council was set up. Although Brazil also extended its muscle across to Africa and in selected multilateral for such as the WHO, the World Intellectual Property Organization, and the World Trade Organization, as Riggirozzi (2014b) shows health also became a strategic policy driver redefining the terms of regionalism in South America. This unfolds, as Riggirozzi argues, as ‘three levels of policy practice: (1) institutional, as a regulatory actor; (2) diplomatic, engaged in extra-regional relations; and (3) project-led, engaged in intra-regional activities’ (2014b: 14). These levels are interconnected via the exchange of human and economic resources and institutionalization of regulatory frameworks.

**Figure 1.**
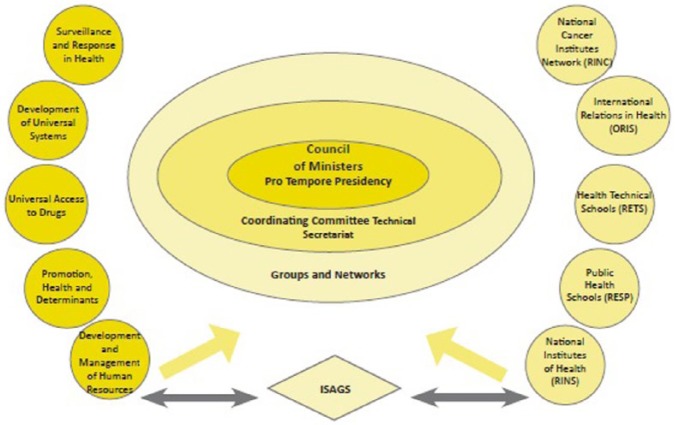
Structure of the South American Health Council. Source: Plan Quinquenal, 2010 – 2015, UNASUR (2010).

The South American Health Council, known more commonly as UNASUR Health, was created in December 2008 with the aim of furthering health cooperation (UNASUR, 2010). The guidelines for cooperation were set out in its 5-year plan (2010–2015) extending to: health surveillance and response; the development of universal health systems; action on social determinants; universal access to medicine; the development of human resources. These five areas were respectively taken up by the five Technical Groups of the Council composed of senior representatives of health ministries. Institutional integration is promoted through the creation of five networks, the National Institutes of Health, the Schools of Public Health, the Schools of Health Technicians, the National Cancer Institutes, and the National Agencies for Health Cooperation. Each of the five networks develops its own agenda for cooperation following the guidelines of the 5-year plan. Beyond that central emphasis for horizontal cooperation, the South American Health Council has a high profile instrumental role in global diplomacy developing common positions in the WHO. Unlike the framework of the European Union where health policy is regulated through supranational institutions (the Commission and Parliament), the Health Council is an intergovernmental body. The creation of the South American Institute of Government in Health (Instituto Sudamericano de Gobierno en Salud or ISAGS) with an important start-up grant from Brazil has allowed continued technical work beyond the intermittent meetings of the Council and its technical groups (see Figure 1). ISAGS is a defining feature in the plethora of new regional initiatives. The organization is hosted in Rio de Janeiro and is meant to open a liason office in Ecuador in order to bring it closer to the UNASUR headquarters and increase its political leverage.

UNASUR’s health drive contributes to promote health as a human right in public policies while it strengthens the hand of South America *vis-à-vis* external actors. Moreover, the South American epidemiological shield in the words of Buss and Tobar (2009) amount to a regional public good, that is, a service which is provided collectively and whose benefits are offered to all groups in the region. A prime example of the importance of the shield was seen at the time of the pandemic influenza. Prior to the eruption of cases in Argentina and Chile, an early warning system triggered joint action at the borders, while countries got going with diagnostic and therapeutic methods. The notion of health as a regional public good in turn leads to the promotion of universal and equitable health systems. The regional value added involves the development of trans-border healthcare and access to the national services in each of the member countries for all inhabitants. This is of no small importance in a region with intense migration flows and where migrants often remain employed informally in host countries (Braga et al., 2011).

To the sanitary challenge, UNASUR added the political challenge of designing regional strategies towards better access to medicines through joint negotiations with pharmaceutical companies to secure fair prices for drugs, diagnostic kits, vaccines and medical equipment, and the improvement of human and industrial capacity. In another manifestation of collective action, UNASUR countries have committed not to buy medicines above the prices settled by PAHO’s fund, attempting to prevent commercial interests taking advantage of panic and the uncertainty caused by epidemics. Patents and access to medicine have demanded a more nuanced assessment of how regional arrangements can maximize and enhanced the reach and outcomes of public policy, emphasizing that economic interests in the global health industry and intellectual property laws should not become an obstacle to protect public health. The promotion of universal access to medicines has been prioritized by establishing new drug policies and the development of a South American health production complex to allow access to medication to vulnerable groups below the poverty line, crucial in addressing the social determinants, an ambitious aim considering the high stakes of pharmaceutical multinationals in retaining the power of monopoly pricing policies. Although the realm is peppered with power, interests, and value conflicts, it is actually within reach. It should be noted that Argentina has strengthened its public production and Brazil is well known for its production of generic medicines.

At this point, the creation of ISAGS stands out. It is a nimble organization with an annual budget below US$3 million. It brings together an innovative network of health ministers of member countries, academics, health specialists, and technicians with the aim of supporting and strengthening national and sub-regional capacity in the formulation, implementation, and evaluation of policies and long-term plans. Headquartered in Rio de Janeiro, ISAGS meets the needs for training human resources in response to the targets of the 5-year plan and in response to the need to train leaders in the formulation of health policies. ISAGS leads a network of similar country-based institutions dedicated to the production of knowledge and preparation of key professionals for the management of national health systems. The establishment of ISAGS is a pioneering step. It is an institutional pillar to tackle issues of management and redistribution of resources in the form of human capacity for better governing of health as a regional goal as well as professionally for enhancing research and development^2^ (Riggirozzi, 2014a). ISAGS activities range from the organization of seminars, courses, internship programs, and other initiatives to improve management of health systems, to the coordination of research initiatives in support of a more autonomous pharmaceutical industry. In this respect, ISAGS seeks to identify existing industrial capacities in the region to coordinate common policies for production of medicines and other goods, advancing the industry and creating competitive advantages in global negotiation and provision for regional health. UNASUR Health institutions, both the Council and its technical groups hand in hand with ISAGS, are crucial mechanisms for establishing a normative framework. While the Health Council essentially organizes the rules and procedures fostering relationships between actors, ISAGS acts as a regional think tank aiming to redefine boundaries between public interests and private actors and broker the ensuing tensions the technical issues. Also has a normative advocacy role on a range of global public health issues; and between institutional commitments of ‘health for all’ and the interests and influences of the health private sector.

UNASUR’s flag in global health is the promotion of policies related with rights to health and universal access. One of the first positions taken by UNASUR at the WHO was concerning the impact of intellectual property rights on access to medicines and the monopolist position of pharmaceutical companies on price setting and generics (Riggirozzi, 2014a). At the 65th World Health Assembly in May 2012, a resolution proposed by UNASUR and led by Ecuador and Argentina was approved asking for an intergovernmental group to replace the International Medical Products Anti-Counterfeiting Taskforce (IMPACT) – an agency led by Big Pharma and the International Criminal Police Organization (Interpol) – to act on, and prevent, counterfeiting of medical products (Riggirozzi, 2014a). What is more, for the first time, joint actions are being promoted at the PAHO and the WHO to change policies regarding representation of developing countries in the executive boards of these bodies. Likewise, UNASUR governments have set out a strategy of joint negotiation through PAHO’s revolving fund to guarantee equitable access to medicines. Yet UNASUR is especially wary of the intrusion of external donors dictating global health programs and particularly of glossy NGOs, such as the Bill and Melinda Gates Foundation, its financial muscle, and influence on the agendas of health institutions. The concern is that the increasing gravity of voluntary contributions restricts WHO’s ability to fully plan its operations or present a core strategy. Even more crucially, the concern is that the WHO is ever more reliant on the priorities of such large donors and decreasingly an independent organization. UNASUR has been forthrightly vocal in protecting the role of states in the governance of WHO.

One of the most salient actions of heightened health diplomacy was the cooperation with Haiti after the devastating earthquake in January 2010. The 12 members of UNASUR disbursed US$70 million, out a total of US$100 million committed to the reconstruction of Haiti. These funds are allocated for the implementation of 144 projects identified, coordinated, and funded by UNASUR. They aimed to strengthen the Haitian state, and specifically with regard to its ability to act in the health area, to deliver and install healthcare equipment, and to train professionals at all levels, UNASUR provided relief supplies to assist counter-cholera efforts targeting Haiti’s dire sanitation crisis after the earthquake. Under the coordination of UNASUR, Venezuela sent medical supplies to help combat the outbreak of cholera in the island. Likewise, a mission also undertook an extensive vaccination against H1N1 influenza and dengue. Additional bilateral aid from Cuba, Ecuador, and Dominican Republic provided support in the form of funds, logistics, sanitation, and personnel, reinforcing the regional response. Cooperation with Haiti has also complemented health assistance with food sovereignty and improvement of infrastructure, housing, and institutional strengthening. The Health Council also played a key role after the earthquake in Chile in 2010.

All told, UNASUR promotes a movement toward horizontal cooperation and technical support, away from what its leaders view as an outmoded vertical model of donors and recipients. Although the idea of social policy through regionalism is hardly ‘new’ insofar as some cross-border projects on health were supported within the structure of both the Andean Community and MERCOSUR, what is new is both content and the ground laid for institutional cooperation. This is a major policy difference with former cooperation in trade-led agreements which promoted pro-market provision within countries. In such agreements health at most enjoyed a residual status; the agenda focused on sanitary security rather than health promotion as such. Building on those stepping stones but in a more assertive political environment, UNASUR embraced health, both as integral parts of cooperation and as a part of the rights agenda. Health took centrality not only as a sanitary problem of trans-border relations but also and fundamentally as a right to be sought in intra-regional relations and in global governance diplomacy (Buss, 2011). The building blocks of regional health diplomacy would then comprise norm creation in public health forums, the epidemiological shield, governance of global health, policy and strategy development, support to health systems, and human resource development. This overall approach within an institutional setting is what Almeida (2011), Buss (2011), and Ventura (2014) describe as ‘structured cooperation’.

UNASUR became a game changer in regional diplomacy. In a region marked by poverty and social inequalities, these remain major causes of health problems or the prime cause of causes. This in a nutshell is addressed by the notion of social determinants of health. UNASUR understands health as part and parcel of social inequalities and as a human right for which the right governance mechanisms need to be laid out.

## Conclusion

UNASUR was born out of the perceived need to counterbalance the US agenda of market-led reform forcefully endorsed by World Bank and Inter-American Development Bank loans which together with the Free Trade Area of the Americas prodded countries at a time of financial need into providing access to private business. The inception of UNASUR is part of the new cycle of President-led, post-neoliberal, post-hegemonic regional cooperation sidelining financial and trade conditionality packages. In 2008, it emerged as a very high profile and high politics exercise at a moment of intensely personalized presidential diplomacy. While high profile issues such as defense and monetary cooperation were riddled with conflicts and faced numerous obstacles, remarkably health cooperation moved with the times and acquired a life of its own at the technical level, somewhat above the political fray, expressing the building blocks of a left that is sensitive to international issues and solidarity beyond the nation state. Slowly but persistently, a novel kind of diplomacy reaching out to the needs of peoples moved on. For one thing, programs against hunger were able to reduce undernourishment and also cut in half the proportion of the population going hungry. For another, UNASUR Health built on the global momentum provided by the rise to prominence of global health diplomacy. Bent on trying to capture social demands, and banking on the networks of health scientists, it is a prominent example of a shared awareness and appreciation of the value of the public good which health is, as well as a will for bilateral, regional, and multilateral cooperation.

The degree of foreign policy attention devoted today to social issues and health, in particular, is historically unprecedented and amounts to a quiet revolution way beyond the XIX century start to counter transboundary health issues or the customs-based focus of the XX century. From those early beginnings in sanitary cordons or customs regulations, it has moved to the promotion of health as a human right. From the concern with epidemics it has moved to mutual cooperation for the overall strengthening of national health systems. ISAGS is a pioneering institution both in terms of being an agile knowledge bank and a training center. Healthcare is obviously not free from political and tactical use. While presidents can get credit from their constituents for practicing altruist leadership, the *unasurization* of health policies, understood as the process of building a new health framework (Herrero, 2015) is not devoid of conflict of interests and values. Without even a trace of intending a one size fits all approach, cooperation faces numerous challenges in a region where business actors fiercely resist change and the social determinants of health prevail, an issue that opens up to income disparities, gender, nutrition, clean air, clean water. While poverty reduction is at the forefront of development in South America and much has been achieved over the last decade with conditional cash transfers and active social policies, countries still face a double burden of disease.^3^

The handmaiden for region building in the 1990s was trade; the 21st century opens with a social turn and healthcare in focus. Unfortunately, the emergence of new players, resources, and political support for global health has yet to deliver tangible change in health outcomes for all populations. The current transboundary health challenges are evident and UNASUR’s agenda is ambitious. The first steps underline the need to build capacity for global health diplomacy by training public health professionals to face the challenges ahead and diplomats to understand the value of the joint creation of health frameworks which take time and seldom reach the headlines. UNASUR offers an opportunity structure for both diplomats and public health professionals, to see health as a key issue for horizontal cooperation in multilateral and national fora and make progress towards a shared framework through networking, capacity sharing, and capacity building. UNASUR offers a privileged site for projecting a foreign policy of good causes that flags values and identities in ways that seem unparalleled by any other extant regional organization in South America or beyond. Regional health diplomacy is a field in the making which by tackling the social determinants of health aims to turn health from a humanitarian concern to a human right. Human rights networks have learnt the value amalgamating normative agenda-setting with sound technical knowledge. There is of course no guarantee that health will retain the center stage in foreign policy that it has held over the last decade or that it can avoid this pitfall of regional politics. Has the concept of regional health policy taken root? Will it continue to fly? As the Presidents that held these as their pet issues give way to new ones in the course of electoral competition, an impasse can bedevil the organization. For one thing, new types of alliances pop up on the sidelines and reduce coherence. Some of the member countries have switched allegiances to the Pacific Alliance, clearly anchored on trade and averse to radical thinking. For another, domestic political strife in Venezuela together with the gradual enfeeblement of President Dilma Roussef in Brazil has by force eroded the political drive for regional social policy making and indeed of social radicalism. The tide can certainly turn and strategic direction can decline, but if the right to health is not to lose traction, UNASUR’s political arm can rely on its technical arm to continue functioning, and the latter can provide capital for political leverage and leadership policymakers need to embed these efforts and explore the unique interface between the theory and the practice of international relations in the field of health. Civil society as a powerful protagonist will be key to assisting with this process holding on to the momentum as it has done in the field of human rights more broadly and so very particularly in South America.

## Notes

1. The Oswaldo Cruz Foundation was founded in 1900 by Dr Oswaldo Cruz, a noted physician and epidemiologist. Today it is considered one of the world’s main public health research institutions. It has laboratory testing capacity for Ebola. It is also a vaccine-manufacturing center with considerable export capacity, selling to PAHO, Argentina, Paraguay.

2. This draws largely from Riggirozzi (2014a).

3. While chronic diseases such as diabetes, heart diseases, and cancer increase and many nations still battle tuberculosis, pneumonia, diarrhea, and malaria and struggle to reduce maternal and child deaths caused by infectious diseases (WHO, 2013).
